# Linking Diabetes to Alzheimer’s Disease: Potential Roles of Glucose Metabolism and Alpha-Glucosidase

**DOI:** 10.2174/1570159X21999221111102343

**Published:** 2023-08-15

**Authors:** Ai Sze Wee, Thao Dinh Nhu, Kooi Yeong Khaw, Kim San Tang, Keng Yoon Yeong

**Affiliations:** 1 School of Pharmacy, Monash University Malaysia, Bandar Sunway, 47500, Selangor, Malaysia;; 2 Faculty of Medicine, SEGi University, Kota Damansara, 47810 Selangor, Malaysia;; 3 Faculty of Pharmacy and Pharmaceutical Sciences, Monash University, 381 Royal Parade, Parkville, VIC 3052, Australia;; 4 School of Science, Monash University Malaysia, Bandar Sunway, 47500 , Selangor, Malaysia;; 5 Tropical Medicine and Biology (TMB) Multidisciplinary Platform, Monash University Malaysia, Bandar Sunway 47500 Selangor, Malaysia

**Keywords:** Alpha-glucosidase, Alzheimer’s disease, amyloid beta-peptides, apolipoprotein E, diabetes mellitus, hyperglycaemia, tau proteins

## Abstract

Alzheimer’s disease (AD) and type 2 diabetes mellitus (DM) are more prevalent with ageing and cause a substantial global socio-economic burden. The biology of these two conditions is well elaborated, but whether AD and type 2 DM arise from coincidental roots in ageing or are linked by pathophysiological mechanisms remains unclear. Research findings involving animal models have identified mechanisms shared by both AD and type 2 DM. Deposition of β-amyloid peptides and formation of intracellular neurofibrillary tangles are pathological hallmarks of AD. Type 2 DM, on the other hand, is a metabolic disorder characterised by hyperglycaemia and insulin resistance. Several studies show that improving type 2 DM can delay or prevent the development of AD, and hence, prevention and control of type 2 DM may reduce the risk of AD later in life. Alpha-glucosidase is an enzyme that is commonly associated with hyperglycaemia in type 2 DM. However, it is uncertain if this enzyme may play a role in the progression of AD. This review explores the experimental evidence that depicts the relationship between dysregulation of glucose metabolism and AD. We also delineate the links between alpha-glucosidase and AD and the potential role of alpha-glucosidase inhibitors in treating AD.

## 1 INTRODUCTION

Alzheimer’s disease (AD) has been identified as the fifth leading cause of death among the elderly [1]. In the worldwide population, it is estimated that more than 40 million people suffers from AD and this figure is projected to increase considerably [2]. AD is characterised by impairment of cognitive function as well as the presence of neuropathological biomarkers, which include the well reported aggregation of insoluble β-amyloid (Aβ) and neurofibrillary tangles containing phosphorylated tau protein [3]. During the progression of AD, Aβ peptides have often been shown to be deposited in the brain. This fundamental change makes Aβ aggregation an early event in the pathogenesis of AD. It is also postulated that Aβ deposition leads to the generation of neurofibrillary tangles, and eventually neuronal death. The burden of AD on the nation’s health care system and the caregivers is substantial. Hence, there is a crucial need for disease-modifying therapies that could slow down the rate of disease progression or prevent the occurrence of this disease. Unfortunately, the available therapeutic options for AD are only modestly effective, and this might result from the fact that the cause of the disease is not fully understood and yet to be fully elucidated.

Interestingly, hyperglycaemia, hyperinsulinemia, hypertension, hyperlipidaemia and obesity have been associated with an increased risk of late-onset AD [4-7]. A study conducted by Xu *et al.* (2007) revealed that borderline diabetes mellitus (DM) due to impaired glucose regulation increased the risk of developing AD [8]. Moreover, many studies have identified DM as a risk factor for AD [9-11]. Several studies have shown that individuals with type-2 DM are more likely to develop AD [12, 13]. Similarly, increased risk of developing dementia has been tied to individuals with high blood glucose levels [14, 15]. Furthermore, those with elevated blood glucose levels also noted a faster conversion from mild cognitive impairment (MCI) to AD [16, 17]. This suggests that AD pathogenesis could have a connection with disrupted glucose homeostasis.

The association between type-2 DM and AD is complex, and both are interlinked with similar underlying mechanisms including insulin resistance, insulin growth factor (IGF) signalling, inflammatory response, oxidative stress, glycogen synthase kinase 3β (GSK-3β) signalling mechanism, cholinergic impairment, Aβ aggregation, neurofibrillary tangle formation, among others [18]. Owing to the socio-economic impacts of DM and AD, understanding the interplay between these two diseases is imperative. The increased risk of AD in DM has been highlighted in several studies to involve the dysfunction of insulin signalling-related mechanisms [19-21].

Alpha-glucosidase plays an essential role in the regulation of blood glucose, and the inhibition of this enzyme could suppress postprandial hyperglycaemia. Whether administered alone or in combination with other anti-diabetic drugs, USFDA approved alpha-glucosidase inhibitors (AGIs), have been reported to be particularly useful for the treatment of type 2 DM [22, 23]. Although AGIs have shown some benefits in type 1 DM, gestational DM, and decreasing body weight, they are not approved by FDA for these indications [24]. In a study conducted by Zhang *et al.* (2016), acarbose has been reported to reduce the postprandial glucose level. It was also found that homeostatic model assessment of β-cell function (HOMA-β) was also reduced significantly in subjects with lower baseline HbA1c. Moreover, they also found that acarbose is able to reduce triglyceride, insulin and glucagon more than metformin at all HbA1c levels [25]. In this article, we aim to review the clinical and experimental findings linking hyperglycaemia, cognitive function, and hallmark markers of AD. The potential of repurposing α-glucosidase enzyme inhibitors to reduce the risk of AD is also discussed in this review.

## 2 HYPERGLYCAEMIA AND AD

**Fig. (1):**
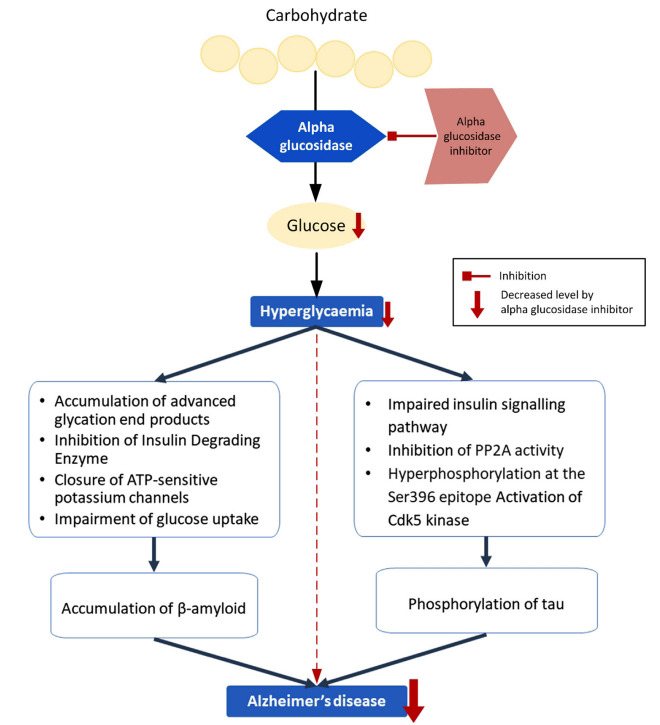
Mechanisms underlying hyperglycaemia-induced accumulation of β-amyloid and increased phosphorylation of tau.

The prevalence of type 2 DM and AD is increasing in the ageing population, and there is evidence demonstrating that hyperglycaemia is a potential risk factor for the development of AD (Fig. **1**). These factors are discussed in detail below.

### 2.1 Hyperglycaemia-Induced Accumulation of Aβ

A decrease in insulin production by beta islet cells and the impairment of insulin receptors are among the factors that lead to hyperglycaemia. Abnormal accumulation of advanced glycation end products (AGEs) due to hyperglycaemia has been demonstrated to increase the production of reactive oxygen species (ROS), which in turn stimulates downstream APP-related pathway, Aβ production [26], NAD+-dependent deacetylase sirtuin 1 (Sirt1) and glucose regulatory protein 78 (GRP78). This subsequently upregulates cell death related pathways in neuronal cells, leading to development of AD [27]. In addition, AGEs are thought to be neurotoxic as they reduce cell viability in primary cortical neurons [28].

Aβ is generated from APP through cleavage by β-secretase (β-site APP cleaving enzyme, known as BACE) and γ-secretase (presenilin complex comprising PS1 and PS2). Yang *et al.* (2013) reported that hyperglycaemia-induced Aβ production is due to inhibited APP degradation in neuronal-like and non- neuronal cells [29]. Ample evidence has shown that abnormal insulin signalling in brain insulin resistance enahnces Aβ accumulation in animal models of type 1 and type 2 DM. Sajan *et al.* (2016) revealed that hyperinsulinemia increases in activities of Akt and atypical protein kinase C in the brains of insulin-resistant mice and monkeys, resulting in elevated Aβ levels [30]. Currais *et al.* (2012) demonstrated that streptozotocin (STZ)-induced T1DM was accompanied by higher levels of Aβ, APP, and tau phosphorylation in the hippocampus of senescence-accelerated mice [31]. Similarly, STZ injection-induced T1DM not only aggravated Aβ accumulation but also upregulated both full-length APP and beta-site APP cleaving enzyme 1 [32-34]. In addition, STZ-induced diabetic rats showed hippocampus atrophy, synapse loss in the brain, Aβ aggregation, and impaired performance of memory and learning functioning [35].

In addition, insulin-degrading enzyme (IDE) causes degradation of Aβ in both *in vitro* and *in vivo* models [36-38]. Qiu *et al.* (1998) and Vekrellis *et al.* (2000) have suggested that insulin influences IDE in the clearance of Aβ in AD patients [39, 40]. Studies have demonstrated that insulin increases extracellular Aβ 1-40 and Aβ 1-42 levels in neuroblastoma SH-SY5Y cells and primary cultures of rat cortical neurons. Insulin not only inhibits the extracellular Aβ degradation by IDE but also stimulates Aβ secretion, which results in a significant reduction of Aβ1-40 and Aβ1-42 intracellular concentrations [41-43].

Acute hyperglycaemia is suggested to elevate the hippocampal interstitial fluid (ISF) Aβ levels by inducing changes in neuronal activity [44]. Several *in vivo* studies have demonstrated that increased synaptic activity can drive Aβ release from an endocytic pool to result in increased ISF Aβ levels [45-47]. In addition, hyperglycaemia-induced rapid neuronal excitability may involve ATP-sensitive potassium (K_ATP_) channels, in which closure of these channels can lead to a rise in extracellular β-amyloid concentration.

The glucose transporter (GLUT) proteins, as the name suggests are proteins which are involved in transporting glucose to different locations in our body The function of GLUT proteins (GLUT1-4) can be regulated by the IGF family. Insulin/IGF-1 signaling pathway can mediate neuronal excitability, metabolism, and survival. Any abnormality or disruption in this pathway may trigger continuous dwindling of neurons in AD brains [48, 49]. In addition, altered neuronal IGF-1 function can contribute to the neuronal pathology and overall synaptic caused by APP- Aβ clearance in the apolipoprotein E (APOE) ε carriers, thus possibly leading to increased neuritic plaque formation in AD [50]. Meanwhile, the malfunctioning of GLUT4 protein in the hippocampus can alter the cognitive flexibility and biochemical reactions in this brain area, posing a risk of developing depression and impaired cognitive function, in which might contribute to AD development [51, 52].

A study conducted by An *et al.* (2018) proposed that AD progression may occur with abnormalities in brain glucose homeostasis. The subtle changes may already start years before symptoms are detected clinically [53]. Research has revealed a significant reduction in the activities of glycolysis rate-controlling enzymes - hexokinase, phosphofructokinase and pyruvate kinase, in the inferior temporal gyrus and middle frontal gyrus of individuals with AD, which is also associated with Aβ pathology. They have also reported significant lower protein levels of the neuronal GLUT3 in AD models [53], which are linked with more serious neuritic plaque. Furthermore, GLUT3 protein levels and their association with AD pathology are not linked to neuronal loss as they remain prominent even after adjusting the levels of neuronal nuclear protein nesprin-1 [54]. This demonstrated that lower GLUT3 protein levels in AD are likely to reflect early progression of AD pathophysiology rather than downstream consequences of neurodegeneration. Hence, failure to utilise neuronal glucose due to impaired glycolysis is a fundamental hallmark of AD.

Tharp *et al.* (2016) showed that an increased Aβ secretion in stromal vascular cells (SVC) cultured at high glucose concentration without correlation to APP system transcription [55]. Studies have shown that endogenous Aβ in cerebrospinal fluid and blood may fluctuate widely depending on glucose and insulin [56-58]. Particularly, glucose and insulin can affect the secretase activity, exocytosis pathways, unfolded protein response or induce inflammation and mitochondrial dysfunction, causing adipose tissue cells to secrete Aβ. Additionally, Aβ and insulin are competing for insulin receptors and degradation by IDE, which probably reduces signal transduction through the insulin receptor signaling pathway and prolongs Aβ half-life [59, 60].

One of the features of AD patients is the impairment of glucose uptake in the brain regions containing neuritic plagues [61-63]. A few studies showed that the impairment in glucose uptake and suppression of mitochondrial production of ATP resulting in an increased vulnerability to excitotoxic calcium overload, which was the mechanism of cell injury in the pathogenesis of AD [64-66]. A study by Mark *et al.* (1997) demonstrated that Aβ impaired glucose uptake in cortical neurons and cultured hippocampal *via* mechanism involving 4-hydroxynonenal (HNE) [67]. Aβ-induced impairment of glucose transport preceded the decrease in cellular ATP levels, suggesting the reduced glucose uptake was in correlation to ATP depletion. HNE binds to membrane proteins such as Na^+^/K^+^-ATPase and Ca^2+^-ATPase that are involved in the transport of ions, glutamate and glucose and impairs their function [68-70]. The impairment of the Na^+^/K^+^-ATPase leading to membrane depolarization and promotes Ca^2+^ influx through voltage-dependent channel and NMDA receptors. The impairment of glutamate transport leading to overstimulated glutamate receptor by excessive accumulation of extracellular glutamate. Impairment of glucose transport results in ATP depletion, increased oxidative insults and vulnerability to excitotoxic. As a result of inducing ATP depletion, Aβ altered protein phosphorylation reactions mediated by kinases. For instance, Aβ and metabolic/excitotoxic insults altered the phosphorylation of various cytoskeletal proteins, including the microtubule-associated protein tau [71-74].

### 2.2 Hyperglycaemia-induced Tau Protein Phosphorylation

Phosphorylated tau, a key constituent of paired helical filaments in the AD neurofibrillary tangles, has been linked with deficient insulin signalling in diabetic brains [75, 76]. GSK-3β plays a crucial role in the phosphorylation of tau proteins and insulin signalling. Thus, insulin signalling impairment in the DM that regulates the GSK-3β pathway may increase the AD risk byelevating the phosphorylation of tau proteins.

Tau protein is one of the major microtubule-associated proteins. The interaction between tau and microtubules on a single microtubule-binding site is within millisecond *via* a kiss-and-hop mechanism. It is a highly dynamic interaction that takes place in the densely packed axonal microtubule array [77]. Tau phosphorylation can be modulated by numerous kinases, including GSK-3β, mitogen-activated protein kinase/extracellular-signal-regulated kinase (MAPK/ ERK), c-Jun N-terminal kinase (JNK), and cyclin-dependent kinase 5 (Cdk5). Meanwhile, the main phosphatase for modulating tau phosphorylation is protein phosphatase 2A (PP2A) [78]. Abnormal tau aggregation leads to the generation of neurofibrillary tangles and paired helical filaments (PHFs), a neuropathological hallmark in the brains of patients diagnosed as tauopathies, As DM brains also see a rise in hyperphosphorylated tau, it is often considered a tauopathy-associated disease [79-81]. An *in vitro* study showed that, transfected PC12 cells with pseudophosphorylated tau induces neurodegeneration [82]. Meanwhile, an inducible pseudophosphorylated tau mouse model was generated to investigated the effect of conformational modified tau by Di and colleagues [83]. It is showed that abnormally hyperphosphorylated tau causes neurodegeneration resulting in cognitive deficits. As such, inhibiting the formation of hyperphosphorylated tau can reverse the development of cognitive dysfunction in DM [84]. In addition, Schubert *et al.* (2003) reported increased neurofibrillary tangles containing hyperphosphorylated tau in the hippocampus of IRS-2 knockout mice, typical pathological signs of T2DM [85]. Therefore, impaired insulin signalling along with hyperglycaemia may induce tau phosphorylation and subsequent cleavage, contributing to the increased risk of AD in diabetic patients.

Several studies have revealed that neurodegeneration mediated by the formation of hyperphosphorylated tau contributes to the DM-associated cognitive deficit [86, 87]. Different tau phosphorylation sites were uncovered although intra-cytoplasmic tau-positive tangle-like inclusions or tau aggregation were not detected in the brains of DM patients [88, 89]. Miller *et al.* (2006) revealed an increase of tau phosphorylation at residues such as Thr181, Ser199, Ser202, Thr211, Thr231, Ser262, and Ser396/404, in the hippocampus of mice after STZ treatment for three days [88]. An *in-vivo* study showed a mild increase of tau phosphorylation at PHF-1 (Ser396/404) and AT8 (Ser202 and Thr205) epitopes after 30 days of STZ injection. However, a high increment of tau phosphorylation was detected after 40 days [87]. In other studies, tau phosphorylated at Ser199/Thr202 or Ser396 site was detected in STZ-induced diabetic mice for up to 45 days, respectively [90, 91]. An *in-vivo* study revealed that the degree of tau phosphorylation was well correlated with the degree of cognitive dysfunction and plasma glucose levels. Besides, Wu *et al.* (2017) found a sharp increase in p-S6K and p-mTOR levels in the hippocampus of STZ-induced diabetic rats and the increase correlates with the levels of p-tau [92]. Wang *et al.* (2014) revealed that the inhibition of the over-activated mTOR/S6K signalling with rapamycin rescues cognitive deficits and reverses tau phosphorylation in the same animal models [90].

Tau undergoes several post-translational modifications including phosphorylation [93, 94]. Caspases [95], the ubiquitin-proteasome system [96] and calpains [97, 98] are all indications of tau cleavage, with the formation of various tau fragment sizes during neuronal apoptosis [99-102]. For instance, N- and C-terminal fragmentation induces toxic tau aggregation in N2a cells [103]. Tau cleaved at Asp421 is identified in AD brains [104]. Cleavage at Asp421 by caspase 3 allows tau to assemble more drastically into tau filaments *in vitro* [105], suggesting that cleaved tau could enhance polymerisation kinetics and serve as a nucleation centre, promoting the pathologic assembly of tau filaments [106]. Interestingly, the proportion of tau cleaved at the caspase-3 site increases the dwell time during the kiss-and-hop interaction with microtubules, hindering the axonal transport and initiating region-specific dendritic atrophy in cornu ammonis region 1 pyramidal neurons of the hippocampus [107]. In the absence of tau mutation, truncated tau can facilitate neurofibrillary tangles formation *in vivo* [108]. Furthermore, truncated tau induces apoptosis of cortical neurons *in vitro* [109] and, when expressed in transgenic animals, results in reduced spatial memory and impaired reflexes [110, 111].

Kim *et al.* (2013) showed that hyperglycaemia is one of the contributing factors in tau modification in both *in vitro* and *in vivo* DM models. The study revealed that the extent of tau cleavage depended on time and glucose concentration, and was well-correlated with the presence of cleaved caspase-3 and apoptosis. In fact, hyperglycemic conditions in DM (along with IR in T2DM) may trigger the apoptotic response, including caspase activation, leading to tau cleavage and making neurons more susceptible to Aβ insults. Furthermore, glucose and Aβ might concomitantly facilitate the apoptotic pathway, which generates more toxic tau cleavage through a positive feedback mechanism [112].

PP2A inhibition could be attributed to increased tau phosphorylation [113]. Since PP2A dephosphorylates the tau protein at all its epitomes, PP2A dysregulation can be detrimental. Hypothermia is a common sign of severe hypoglycaemia in DM patients [87]. In hypothermic brain, the change in glucose metabolism can suppress PP2A activity, resulting in abnormal tau phosphorylation. In accordance, another study showed that tau phosphorylation and PP2A inhibition occur in non-obese diabetic (NOD) mice, a model of type 1 DM [81].

STZ-induced Hyperglycaemic mice had increased tau phosphorylation, specifically at the Ser396 epitope. The Ser396 epitope phosphorylation is closely associated with tau pathology [114]. Tau protein levels were higher in the hyperglycemic group compared to the control. Interestingly, reference memory errors for the hyperglycemic mice were greater compared to the control group [86].

Guo *et al.* (2016) showed that hyperglycaemia particularly affected Cdk5 kinase which can be usually activated through the activator proteins p25 and p35. In the Pdx1^+/−^ mice, phosphorylated and steady-state protein levels of Cdk5 and p25 were significantly increased, suggesting that this kinase activation can enable changes in tau phosphorylation [115]. In addition, the activation of these kinases - ERKs, JNKs, and p38 MAPK which all belong to MAPK serine-threonine kinase group [116, 117], has been demonstrated to promote tau phosphorylation and in turn, AD pathophysiological alterations [118].

### 2.3 Expression of APOE4

The three major alleles of human APOE gene, namely ε2, ε3 and ε4, has the frequency of 8.4%, 77.9% and 13.7%, respectively. However, a significant increase (approximately 40%) of the ε4 allele frequency was observed in AD patients [119]. The *APOE ε4* allele increases the risk of developing AD [120]. In asymptomatic APOE ε4 carriers, increased systemic insulin resistance is associated with higher tau phosphorylation [121]. Moreover, there is a strong connection between the *APOE ε4* allele and the comorbidity of AD and DM [122].

A study shows that APOE genotypes affect the formation of senile plaques due to abnormal deposition of Aβ, and also lead to development of cerebral amyloid angiopathy (CAA) [119]. In the AD brains, immunohistological results review the co-deposition of APOE within the senile plaques [123]. Moreover, senile plaques has been shown to be more abundant in *APOE ε4* carriers (40.7%), particularly patients aged between 50 to 59 years [124, 125]. In addition, *APOE ε4* is closely related between CAA and CAA-related haemorrhages [126, 127].

The formation of senile plaques, neurofibrillary tangles and CAA were similarly observed in DM *APOE ε4* carriers [128]. This can be explained by the fact that conditions such as hyperglycaemia, hyperinsulinaemia, insulin resistance, as well as the presence of *APOE ε4* allele, could strongly induces the formation of senile plaques in DM patients [129]. In addition, research also demonstrated that *APOE ε4* carriers have higher risk of susceptible to blood-brain barrier breakdown and reduction in the length of small vessels [130], which could be the causative event in the pathogenesis of AD.

Neuronal damage in the brain caused by chronic neuroinflammation has a significant implication in AD pathogenesis [131]. APOE colocalizes with senile plaques and microglia, suggesting a role for APOE in AD-associated innate immune response. Study found that absence of APOE gene in mice had an increased inflammatory response following treatment with Aβ [132]. However, there is mounting evidence that three isoforms of APOE (apoE2, apoE3 and apoE4) differentially regulate the innate immune response [133]. Not only affecting the deposition of Aβ, *APOE ε4* is also associated with a more robust pro-inflammatory response, which might further worsen the progression of AD. In a comparison study conducted by Lynch *et al.* (2003), they found that APOE4-TR mice expressed higher levels of pro-inflammatory cytokines than APOE3-TR mice following injection of lipopolysaccharide [134]. Furthermore, Ringman *et al.* (2012) demonstrate increased inflammatory response in young *APOE ε4* carriers, which may increases the risk of developing AD later in life [135].

Adult hippocampal neurogenesis plays a vital role in structural plasticity of mature neurons and brain networks maintenance. The initial claims for the non-existence of adult neurogenesis in the mammalian brain have been challenged, and recent studies have drawn opposite conclusions [136, 137]. A study conducted by Jiménez *et al.* (2019) identified the existence of immature neurons in the dentate hippocampal gyrus of healthy people aged 43-87 years, and they have also found that continued neurogenesis was reduced in the dentate gyrus of patients with AD [138]. Impairment in hippocampal neurogenesis resulting from early disease manifestation could increases the risk of developing AD and memory deficits [139]. APOE plays an important role in regulating hippocampal neurogenesis, whereby increased expression of APOE inhibits neural stem or progenitor cell proliferation and maintains the characteristics of these progenitor cells in the dentate region of the hippocampus [140]. In *in vivo* study, APOE4 suppresses hippocampal neurogenesis by interfering with the maturation of hilar γ-aminobutyric acid-containing interneurons, which results in the impaired memory and learning capability [141, 142]. These results are to prove the crucial role of APOE4 in the pathology of dysfunctional neurogenesis that contributes to AD progression.

The loss of synapses is an early pathological characteristic of AD [143]. The three isoforms of APOE were reported to demonstrate different roles in synaptic plasticity and repair mechanisms [144, 145]. In a study conducted by Ji *et al.* (2003), they found that APOE4 dose correlates inversely with dendritic spine density in the hippocampus of AD and healthy aged controls [146]. Moreover, similar observation was reported in an animal study, where reduced dendritic spine density and length were observe in APOE4-TR mice compared with APOE3-TR mice [147]. Interestingly, APOE3 has shown to provide protection against the loss of synapses produced by Aβ oligomers [148]. APOE isoforms demonstrate differential effects on synaptic integrity and dendritic spines. Klein *et al.* (2010) found that excitatory synaptic transmission was reduced in APOE4-TR mice at 1 month, suggesting that APOE4 accounts for functional deficits in amygdala’s early development, which ultimately leads to cognitive disorders later in life [149]. Presence of APOE4 has shown to delay the recycling process of Apoer2, receptor that is found in the synaptic gap between nerve cells. On top of that, APOE4 reduces the function of N-methyl D aspartate receptor which responsible for maintaining the synaptic plasticity [150]. Taken all these observations together, it is undoubtedly that pathogenic effects of APOE4 on the synaptic function is a strong risk factor for the development of AD.

## 3 THE POTENTIAL CONNECTION BETWEEN ALPHA-GLUCOSIDASE AND AD

The link between AD and type 2 DM has been thoroughly investigated through evidence from *in vivo* and clinical studies. An increased risk of developing AD and declined cognitive function at a later age are observed from diabetic patients compared to healthy controls [151]. In fact, the high blood glucose level is associated with a heightened risk of dementia [152]. Moreover, as insulin is reported to modulate Aβ degradation [151] and is involved in neuronal function and memory formation, insulin resistance in various brain regions, including the cerebellar cortex and hippocampus, may lead to diabetic encephalopathy [153]. This highlights that if type 2 DM is well-controlled by a good regime, the incidence of AD in the elderly can be managed.

Alpha-glucosidase is a critical enzyme which regulates blood glucose by specifically targeting 1,4-α-glucopyranosidic bonds and subsequently hydrolysing them to produce α-glucose [154]. Glucosidase enzyme inhibitors, such as acarbose, voglibose, and miglitol, are among the clinically used anti-diabetic agents. The drugs inhibit the alpha-glucosidase enzyme that hydrolases carbohydrates into α-glucose in the small intestine, thus delaying glucose absorption into the bloodstream and suppressing postprandial hyperglycaemia [155]. A study conducted by Hagedoorn *et al.* (2007) reported that the therapeutic effects of alpha-glucosidase inhibitors are due to both the slowed digestion of carbohydrates, and also the fermentation of colonic starch [156]. Interestingly, the connection between the alpha-glucosidase enzyme and AD is not yet well understood. However, some possible connections can be deduced *via* the action of glucosidase enzyme inhibitors towards the progression of AD in diabetic models. In an experiment by Yan *et al.* (2015) where acarbose (20 mg/kg/d) was administered to SAMP8 mice for six months, attenuation of cognitive decline (better performance of spatial learning and memory), elevated level of acetylated histone H4 lysine 8 (H4K8ac), an increase in insulin and insulin receptors were observed [157]. In addition to the conventional pathophysiology of AD, loss of canonical Wnt signalling has been proved to participate in the progression of AD, whereas activation of Wnt/β-catenin signalling through the LRP6 receptor may promote neurogenesis, synaptic plasticity, and suppress tau phosphorylation and neuroinflammation [158]. Noticeably, α-glucosidase inhibitors, miglitol and voglibose, were found to have a strong affinity towards LRP6 proteins, indicating the potential of modulating Wnt signalling as one of the possible mechanisms for AD treatment [158]. On the other hand, when compared with other anti-diabetic drugs such as metformin and DPP4 inhibitors in observational studies that correlated these treatments with the risk of AD, glucosidase enzyme inhibitors are less significant in lowering the AD risk [153, 159]. This can be explained by the fact that this drug class does not possess direct modulation activity on insulin levels. However, the result might have been compromised as glucosidase inhibitors are less frequently prescribed than other agents, thus quantitatively affecting the comparison and overall outcome. Therefore, more research is needed to confirm the cognition-related effects of glucosidase inhibitors on type 2 DM models.

Although acarbose has shown efficacy in controlling blood glucose level, they are less preferred in type 2 DM treatments due to several side effects such as abdominal pain, flatulence, diarrhoea, and inferior effectiveness in regulating insulin and glucose levels compared to other anti-diabetic drugs. However, in order to achieve normal glucose homeostasis, a combination of various pathways to tackle multiple pathophysiological mechanisms is needed, and α-glucosidase is still among the key targets in treating DM. In fact, alternative glucosidase inhibitors from natural sources are currently under development. Xanthone derivatives such as mangiferin [155] and flavonoids such as quercetin [160] can achieve lower IC_50_ than acarbose in inhibiting alpha-glucosidase, accompanied by considerable anti-diabetic effects in *in vivo* models [161, 162]. In addition, several studies have experimented with natural products on both alpha-glucosidase and acetylcholinesterase inhibition simultaneously to show their significant inhibitory effects on these enzymes, for example, the study of Mohan *et al.* (2017) on Indian medicinal plants. This highlights the dual functions of potential alpha-glucosidase/acetylcholinesterase inhibitors in treating comorbidities of AD and type 2 DM [163]. Moreover, it is interesting to note that the compound 8-β-D-glucopyranosylgenistein with glucosidase inhibitory activity from *Genista tenera*, traditional herbal medicine to treat DM, can act as an anti-amyloidogenic and a new ligand of Aβ oligomers for AD as shown by Silva *et al.* (2015) [164]. Therefore, further development of glucosidase inhibitors derived from natural compounds with better safety and efficacy profile is essential and promising for both AD and type 2 DM treatment. An indirect connection between α-glucosidase and AD can be drawn, in which controlling the blood glucose level by glucosidase inhibition may be associated with a lower risk of progressing AD in the elderly.

## CONCLUSION AND FUTURE DIRECTIONS

Despite significant investments in research efforts and resources in AD, we have yet to identify a disease-modifying therapy that has proven effective in humans. The suggested connection between AD and DM indicates that the latter is a potential risk factor for AD. With detailed analyses, it could well play a genuine role in predicting AD occurrence. Hence, repurposing alpha-glucosidase inhibitors, which are currently being used to treat DM as potential AD therapy, is an exciting strategy to explore. Besides, understanding the intersection between each molecular pathway i may prove essential in the development of future drug targeting AD.

## LIST OF ABBREVIATIONS

AD: Alzheimer’s Disease
AGEs: Advanced Glycation End Products
AGIs: Alpha-Glucosidase Inhibitors
CAA: Cerebral Amyloid Angiopathy
DM: Diabetes Mellitus
GLUT: Glucose Transporter
IDE: Insulin-Degrading Enzyme
IGF: Insulin Growth Factor
MCI: Mild Cognitive Impairment
NOD: Non-Obese Diabetic
ROS: Reactive Oxygen Species
SVC: Stromal Vascular Cells

## References

[1] Wong W. (2020). Economic burden of Alzheimer disease and managed care considerations.. Am. J. Manag. Care.

[2] Dumurgier J., Sabia S. (2020). Epidemiology of Alzheimer’s disease: latest trends.. Rev. Prat..

[3] Long J.M., Holtzman D.M. (2019). Alzheimer disease: An update on pathobiology and treatment strategies.. Cell.

[4] Feringa F.M., van der Kant R. (2021). Cholesterol and Alzheimer’s disease; from risk genes to pathological effects.. Front. Aging Neurosci..

[5] Silva M.V.F., Loures C.M.G., Alves L.C.V., de Souza L.C., Borges K.B.G., Carvalho M.G. (2019). Alzheimer’s disease: risk factors and potentially protective measures.. J. Biomed. Sci..

[6] Kim J., Woo S.Y., Kim S., Jang H., Kim J., Kim J., Kang S.H., Na D.L., Chin J., Apostolova L.G., Seo S.W., Kim H.J. (2021). Differential effects of risk factors on the cognitive trajectory of early- and late-onset Alzheimer’s disease.. Alzheimers Res. Ther..

[7] Luchsinger J.A., Cheng D., Tang M.X., Schupf N., Mayeux R. (2012). Central obesity in the elderly is related to late-onset Alzheimer disease.. Alzheimer Dis. Assoc. Disord..

[8] Xu W., Qiu C., Winblad B., Fratiglioni L. (2007). The effect of borderline diabetes on the risk of dementia and Alzheimer’s disease.. Diabetes.

[9] Faqih N.T., Ashoor A.F., Alshaikh S.A., Maglan A.F., Jastaniah N. (2021). Association of Alzheimer’s disease and insulin resistance in King Abdulaziz Medical City, Jeddah.. Cureus.

[10] Thomas K.R., Bangen K.J., Weigand A.J., Edmonds E.C., Sundermann E., Wong C.G., Eppig J., Werhane M.L., Delano-Wood L., Bondi M.W. (2020). Type 2 diabetes interacts with Alzheimer disease risk factors to predict functional decline.. Alzheimer Dis. Assoc. Disord..

[11] Liu L., Volpe S.L., Ross J.A., Grimm J.A., Van Bockstaele E.J., Eisen H.J. (2021). Dietary sugar intake and risk of Alzheimer’s disease in older women.. Nutr. Neurosci..

[12] Ha J., Choi D.W., Kim K.J., Cho S.Y., Kim H., Kim K.Y., Koh Y., Nam C.M., Kim E. (2021). Association of metformin use with Alzheimer’s disease in patients with newly diagnosed type 2 diabetes: a population-based nested case–control study.. Sci. Rep..

[13] Huang C.C., Chung C.M., Leu H.B., Lin L.Y., Chiu C.C., Hsu C.Y., Chiang C.H., Huang P.H., Chen T.J., Lin S.J., Chen J.W., Chan W.L. (2014). Diabetes mellitus and the risk of Alzheimer’s disease: a nationwide population-based study.. PLoS One.

[14] Barbiellini Amidei C., Fayosse A., Dumurgier J., Machado-Fragua M.D., Tabak A.G., van Sloten T., Kivimäki M., Dugravot A., Sabia S., Singh-Manoux A. (2021). Association between age at diabetes onset and subsequent risk of dementia.. JAMA.

[15] Kirvalidze M., Hodkinson A., Storman D., Fairchild T.J., Bała M.M., Beridze G., Zuriaga A., Brudasca N.I., Brini S. (2022). The role of glucose in cognition, risk of dementia, and related biomarkers in individuals without type 2 diabetes mellitus or the metabolic syndrome: A systematic review of observational studies.. Neurosci. Biobehav. Rev..

[16] Dove A., Shang Y., Xu W., Grande G., Laukka E.J., Fratiglioni L., Marseglia A. (2021). The impact of diabetes on cognitive impairment and its progression to dementia.. Alzheimers Dement..

[17] Morris J.K., Vidoni E.D., Honea R.A., Burns J.M. (2014). Impaired glycemia increases disease progression in mild cognitive impairment.. Neurobiol. Aging.

[18] Kandimalla R., Thirumala V., Reddy P.H. (2017). Is Alzheimer’s disease a Type 3 Diabetes? A critical appraisal.. Biochim. Biophys. Acta Mol. Basis Dis..

[19] Zhang Y., Huang N., Yan F., Jin H., Zhou S., Shi J., Jin F. (2018). Diabetes mellitus and Alzheimer’s disease: GSK-3β as a potential link.. Behav. Brain Res..

[20] Tumminia A., Vinciguerra F., Parisi M., Frittitta L. (2018). Type 2 diabetes mellitus and Alzheimer’s disease: Role of insulin signalling and therapeutic implications.. Int. J. Mol. Sci..

[21] Burillo J., Marqués P., Jiménez B., González-Blanco C., Benito M., Guillén C. (2021). Insulin resistance and diabetes mellitus in Alzheimer’s disease.. Cells.

[22] Cai X., Han X., Luo Y., Ji L. (2013). Comparisons of the efficacy of alpha glucosidase inhibitors on type 2 diabetes patients between Asian and Caucasian.. PLoS One.

[23] Dahlén A.D., Dashi G., Maslov I., Attwood M.M., Jonsson J., Trukhan V., Schiöth H.B. (2022). Trends in antidiabetic drug discovery: FDA approved drugs, new drugs in clinical trials and global sales.. Front. Pharmacol..

[24] Gao X., Cai X., Yang W., Chen Y., Han X., Ji L. (2018). Meta-analysis and critical review on the efficacy and safety of alpha-glucosidase inhibitors in Asian and non-Asian populations.. J. Diabetes Investig..

[25] Zhang J.P., Wang N., Xing X.Y., Yang Z.J., Wang X., Yang W.Y. (2016). Efficacy of acarbose and metformin in newly diagnosed type 2 diabetes patients stratified by HbA1c levels.. J. Diabetes.

[26] Kong Y., Wang F., Wang J., Liu C., Zhou Y., Xu Z., Zhang C., Sun B., Guan Y. (2020). Pathological mechanisms linking diabetes mellitus and Alzheimer’s disease: the Receptor for Advanced Glycation End Products (RAGE).. Front. Aging Neurosci..

[27] Ko S.Y., Ko H.A., Chu K.H., Shieh T.M., Chi T.C., Chen H.I., Chang W.C., Chang S.S. (2015). The possible mechanism of advanced glycation end products (AGEs) for Alzheimer’s disease.. PLoS One.

[28] Sato T., Shimogaito N., Wu X., Kikuchi S., Yamagishi S., Takeuchi M. (2006). Toxic advanced glycation end products (TAGE) theory in Alzheimer’s disease.. Am. J. Alzheimers Dis. Other Demen..

[29] Yang Y., Wu Y., Zhang S., Song W. (2013). High glucose promotes Aβ production by inhibiting APP degradation.. PLoS One.

[30] Sajan M., Hansen B., Ivey R., Sajan J., Ari C., Song S., Braun U., Leitges M., Farese-Higgs M., Farese R.V. (2016). Brain insulin signaling is increased in insulin-resistant states and decreases in FOXOs and PGC-1α and increases in Aβ1-40/42 and phospho-tau may abet Alzheimer development.. Diabetes.

[31] Currais A., Prior M., Lo D., Jolivalt C., Schubert D., Maher P. (2012). Diabetes exacerbates amyloid and neurovascular pathology in aging-accelerated mice.. Aging Cell.

[32] Jolivalt C.G., Hurford R., Lee C.A., Dumaop W., Rockenstein E., Masliah E. (2010). Type 1 diabetes exaggerates features of Alzheimer’s disease in APP transgenic mice.. Exp. Neurol..

[33] Lee H.J., Seo H.I., Cha H.Y., Yang Y.J., Kwon S.H., Yang S.J. (2018). Diabetes and Alzheimer’s disease: Mechanisms and nutritional aspects.. Clin. Nutr. Res..

[34] Devi L., Alldred M.J., Ginsberg S.D., Ohno M. (2012). Mechanisms underlying insulin deficiency-induced acceleration of β-amyloidosis in a mouse model of Alzheimer’s disease.. PLoS One.

[35] Wang J.Q., Yin J., Song Y.F., Zhang L., Ren Y.X., Wang D.G., Gao L.P., Jing Y.H. (2014). Brain aging and AD-like pathology in streptozotocin-induced diabetic rats.. J. Diabetes Res..

[36] Kurochkin I.V. (2001). Insulin-degrading enzyme: embarking on amyloid destruction.. Trends Biochem. Sci..

[37] Farris W., Mansourian S., Chang Y., Lindsley L., Eckman E.A., Frosch M.P., Eckman C.B., Tanzi R.E., Selkoe D.J., Guénette S. (2003). Insulin-degrading enzyme regulates the levels of insulin, amyloid β-protein, and the β-amyloid precursor protein intracellular domain in vivo.. Proc. Natl. Acad. Sci. USA.

[38] Azam M.S., Wahiduzzaman M., Reyad-ul-Ferdous M., Islam M.N., Roy M. (2022). Inhibition of insulin degrading enzyme to control diabetes mellitus and its applications on some other chronic disease: a critical review.. Pharm. Res..

[39] Qiu W.Q., Walsh D.M., Ye Z., Vekrellis K., Zhang J., Podlisny M.B., Rosner M.R., Safavi A., Hersh L.B., Selkoe D.J. (1998). Insulin-degrading enzyme regulates extracellular levels of amyloid beta-protein by degradation.. J. Biol. Chem..

[40] Vekrellis K., Ye Z., Qiu W.Q., Walsh D., Hartley D., Chesneau V., Rosner M.R., Selkoe D.J. (2000). Neurons regulate extracellular levels of amyloid beta-protein via proteolysis by insulin-degrading enzyme.. J. Neurosci..

[41] Gasparini L., Gouras G.K., Wang R., Gross R.S., Beal M.F., Greengard P., Xu H. (2001). Stimulation of β-amyloid precursor protein trafficking by insulin reduces intraneuronal β-amyloid and requires mitogen-activated protein kinase signaling.. J. Neurosci..

[42] Mullins R.J., Diehl T.C., Chia C.W., Kapogiannis D. (2017). Insulin resistance as a link between amyloid-beta and tau pathologies in Alzheimer’s disease.. Front. Aging Neurosci..

[43] Kavanagh K., Day S.M., Pait M.C., Mortiz W.R., Newgard C.B., Ilkayeva O., Mcclain D.A., Macauley S.L. (2019). Type-2-diabetes alters CSF but not plasma metabolomic and AD risk profiles in vervet monkeys.. Front. Neurosci..

[44] Macauley S.L., Stanley M., Caesar E.E., Yamada S.A., Raichle M.E., Perez R., Mahan T.E., Sutphen C.L., Holtzman D.M. (2015). Hyperglycemia modulates extracellular amyloid-β concentrations and neuronal activity in vivo.. J. Clin. Invest..

[45] Stanley M., Macauley S.L., Caesar E.E., Koscal L.J., Moritz W., Robinson G.O., Roh J., Keyser J., Jiang H., Holtzman D.M. (2016). The effects of peripheral and central high insulin on brain insulin signaling and amyloid-β in young and old APP/PS1 mice.. J. Neurosci..

[46] Cirrito J.R., Yamada K.A., Finn M.B., Sloviter R.S., Bales K.R., May P.C., Schoepp D.D., Paul S.M., Mennerick S., Holtzman D.M. (2005). Synaptic activity regulates interstitial fluid amyloid-beta levels in vivo.. Neuron.

[47] Yan P., Bero A.W., Cirrito J.R., Xiao Q., Hu X., Wang Y., Gonzales E., Holtzman D.M., Lee J.M. (2009). Characterizing the appearance and growth of amyloid plaques in APP/PS1 mice.. J. Neurosci..

[48] Carro E., Torres-Aleman I. (2004). The role of insulin and insulin-like growth factor I in the molecular and cellular mechanisms underlying the pathology of Alzheimer’s disease.. Eur. J. Pharmacol..

[49] Hayes C.A., Ashmore B.G., Vijayasankar A., Marshall J.P., Ashpole N.M. (2021). Insulin-Like Growth Factor-1 differentially modulates glutamate-induced toxicity and stress in cells of the neurogliovascular unit.. Front. Aging Neurosci..

[50] Kianpour Rad S., Arya A., Karimian H., Madhavan P., Rizwan F., Koshy S., Prabhu G. (2018). Mechanism involved in insulin resistance via accumulation of β-amyloid and neurofibrillary tangles: link between type 2 diabetes and Alzheimer’s disease.. Drug Des. Devel. Ther..

[51] Dehghan F., Hajiaghaalipour F., Yusof A., Muniandy S., Hosseini S.A., Heydari S., Salim L.Z.A., Azarbayjani M.A. (2016). Saffron with resistance exercise improves diabetic parameters through the GLUT4/AMPK pathway in-vitro and in-vivo.. Sci. Rep..

[52] Sayem A., Arya A., Karimian H., Krishnasamy N., Ashok Hasamnis A., Hossain C. (2018). Action of phytochemicals on insulin signaling pathways accelerating Glucose Transporter (GLUT4) protein translocation.. Molecules.

[53] An Y, Varma VR, Varma S (2018). Evidence for brain glucose dysregulation in Alzheimers dement..

[54] Dammer E.B., Duong D.M., Diner I., Gearing M., Feng Y., Lah J.J., Levey A.I., Seyfried N.T. (2013). Neuron enriched nuclear proteome isolated from human brain.. J. Proteome Res..

[55] Tharp W.G., Gupta D., Smith J., Jones K.P., Jones A.M., Pratley R.E. (2016). Effects of glucose and insulin on secretion of amyloid‐β by human adipose tissue cells.. Obesity (Silver Spring).

[56] Zhang Y., Zhou B., Zhang F., Wu J., Hu Y., Liu Y., Zhai Q. (2012). Amyloid-β induces hepatic insulin resistance by activating JAK2/STAT3/SOCS-1 signaling pathway.. Diabetes.

[57] Fishel M.A., Watson G.S., Montine T.J., Wang Q., Green P.S., Kulstad J.J., Cook D.G., Peskind E.R., Baker L.D., Goldgaber D., Nie W., Asthana S., Plymate S.R., Schwartz M.W., Craft S. (2005). Hyperinsulinemia provokes synchronous increases in central inflammation and beta-amyloid in normal adults.. Arch. Neurol..

[58] Stanley M., Macauley S.L., Holtzman D.M. (2016). Changes in insulin and insulin signaling in Alzheimer’s disease: cause or consequence?. J. Exp. Med..

[59] Yamamoto N., Ishikuro R., Tanida M., Suzuki K., Ikeda-Matsuo Y., Sobue K. (2018). Insulin-signaling pathway regulates the degradation of amyloid β-protein via Astrocytes.. Neuroscience.

[60] Townsend M., Mehta T., Selkoe D.J. (2007). Soluble Abeta inhibits specific signal transduction cascades common to the insulin receptor pathway.. J. Biol. Chem..

[61] Jagust W.J., Seab J.P., Huesman R.H., Valk P.E., Mathis C.A., Reed B.R., Coxson P.G., Budinger T.F. (1991). Diminished glucose transport in Alzheimer’s disease: dynamic PET studies.. J. Cereb. Blood Flow Metab..

[62] Koepsell H. (2020). Glucose transporters in brain in health and disease.. Pflugers Arch..

[63] Rebelos E., Rinne J.O., Nuutila P., Ekblad L.L. (2021). Brain glucose metabolism in health, obesity, and cognitive decline-does insulin have anything to do with it? A narrative review.. J. Clin. Med..

[64] Muddapu V.R., Dharshini S.A.P., Chakravarthy V.S., Gromiha M.M. (2020). Neurodegenerative diseases – is metabolic deficiency the root cause?. Front. Neurosci..

[65] Mark R.J., Pang Z., Geddes J.W., Uchida K., Mattson M.P. (1997). Amyloid beta-peptide impairs glucose transport in hippocampal and cortical neurons: involvement of membrane lipid peroxidation.. J. Neurosci..

[66] Cheng B., Mattson M.P. (1992). IGF-I and IGF-II protect cultured hippocampal and septal neurons against calcium-mediated hypoglycemic damage.. J. Neurosci..

[67] Mark R.J., Lovell M.A., Markesbery W.R., Uchida K., Mattson M.P. (1997). A role for 4-hydroxynonenal, an aldehydic product of lipid peroxidation, in disruption of ion homeostasis and neuronal death induced by amyloid beta-peptide.. J. Neurochem..

[68] Mark R.J., Hensley K., Butterfield D.A., Mattson M.P. (1995). Amyloid beta-peptide impairs ion-motive ATPase activities: evidence for a role in loss of neuronal Ca2+ homeostasis and cell death.. J. Neurosci..

[69] Mattson M.P. (2009). Roles of the lipid peroxidation product 4-hydroxynonenal in obesity, the metabolic syndrome, and associated vascular and neurodegenerative disorders.. Exp. Gerontol..

[70] Keller J.N., Mark R.J., Bruce A.J., Blanc E., Rothstein J.D., Uchida K., Waeg G., Mattson M.P. (1997). 4-Hydroxynonenal, an aldehydic product of membrane lipid peroxidation, impairs glutamate transport and mitochondrial function in synaptosomes.. Neuroscience.

[71] Rajmohan R., Reddy P.H. (2017). Amyloid-beta and phosphorylated tau accumulations cause abnormalities at synapses of Alzheimer’s disease neurons.. J. Alzheimers Dis..

[72] Deng L., Pushpitha K., Joseph C., Gupta V., Rajput R., Chitranshi N., Dheer Y., Amirkhani A., Kamath K., Pascovici D., Wu J.X., Salekdeh G.H., Haynes P.A., Graham S.L., Gupta V.K., Mirzaei M. (2019). Amyloid β induces early changes in the ribosomal machinery, cytoskeletal organization and oxidative phosphorylation in retinal photoreceptor cells.. Front. Mol. Neurosci..

[73] Smith-Swintosky V.L., Pettigrew L.C., Sapolsky R.M., Phares C., Craddock S.D., Brooke S.M., Mattson M.P. (1996). Metyrapone, an inhibitor of glucocorticoid production, reduces brain injury induced by focal and global ischemia and seizures.. J. Cereb. Blood Flow Metab..

[74] Busciglio J., Lorenzo A., Yeh J., Yankner B.A. (1995). β-Amyloid fibrils induce tau phosphorylation and loss of microtubule binding.. Neuron.

[75] Gonçalves R.A., Wijesekara N., Fraser P.E., De Felice F.G. (2019). The link between Tau and insulin signaling: Implications for Alzheimer’s disease and other tauopathies.. Front. Cell. Neurosci..

[76] Rodriguez-Rodriguez P., Sandebring-Matton A., Merino-Serrais P., Parrado-Fernandez C., Rabano A., Winblad B., Ávila J., Ferrer I., Cedazo-Minguez A. (2017). Tau hyperphosphorylation induces oligomeric insulin accumulation and insulin resistance in neurons.. Brain.

[77] Janning D., Igaev M., Sündermann F., Brühmann J., Beutel O., Heinisch J.J., Bakota L., Piehler J., Junge W., Brandt R. (2014). Single-molecule tracking of tau reveals fast kiss-and-hop interaction with microtubules in living neurons.. Mol. Biol. Cell.

[78] Schweiger S., Matthes F., Posey K., Kickstein E., Weber S., Hettich M.M., Pfurtscheller S., Ehninger D., Schneider R., Kraub S. (2017). Resveratrol induces dephosphorylation of Tau by interfering with the MID1-PP2A complex.. Sci. Rep..

[79] Platt T.L., Beckett T.L., Kohler K., Niedowicz D.M., Murphy M.P. (2016). Obesity, diabetes, and leptin resistance promote tau pathology in a mouse model of disease.. Neuroscience.

[80] Jung H.J., Kim Y.J., Eggert S., Chung K.C., Choi K.S., Park S.A. (2013). Age-dependent increases in tau phosphorylation in the brains of type 2 diabetic rats correlate with a reduced expression of p62.. Exp. Neurol..

[81] Papon M.A., El Khoury N.B., Marcouiller F., Julien C., Morin F., Bretteville A., Petry F.R., Gaudreau S., Amrani A., Mathews P.M., Hébert S.S., Planel E. (2013). Deregulation of protein phosphatase 2A and hyperphosphorylation of τ protein following onset of diabetes in NOD mice.. Diabetes.

[82] Alonso A.D., Di Clerico J., Li B., Corbo C.P., Alaniz M.E., Grundke-Iqbal I., Iqbal K. (2010). Phosphorylation of tau at Thr212, Thr231, and Ser262 combined causes neurodegeneration.. J. Biol. Chem..

[83] Di J., Cohen L.S., Corbo C.P., Phillips G.R., El Idrissi A., Alonso A.D. (2016). Abnormal tau induces cognitive impairment through two different mechanisms: synaptic dysfunction and neuronal loss.. Sci. Rep..

[84] Qu Z.S., Li L., Sun X.J., Zhao Y.W., Zhang J., Geng Z., Fu J.L., Ren Q.G. (2014). Glycogen synthase kinase-3 regulates production of amyloid-β peptides and tau phosphorylation in diabetic rat brain.. Sci. World J..

[85] Schubert M., Brazil D.P., Burks D.J., Kushner J.A., Ye J., Flint C.L., Farhang-Fallah J., Dikkes P., Warot X.M., Rio C., Corfas G., White M.F. (2003). Insulin receptor substrate-2 deficiency impairs brain growth and promotes tau phosphorylation.. J. Neurosci..

[86] Zhou Y., Zhao Y., Xie H., Wang Y., Liu L., Yan X. (2015). Alteration in amyloid β42, phosphorylated tau protein, interleukin 6, and acetylcholine during diabetes-accelerated memory dysfunction in diabetic rats: correlation of amyloid β42 with changes in glucose metabolism.. Behav. Brain Funct..

[87] Planel E., Tatebayashi Y., Miyasaka T., Liu L., Wang L., Herman M., Yu W.H., Luchsinger J.A., Wadzinski B., Duff K.E., Takashima A. (2007). Insulin dysfunction induces in vivo tau hyperphosphorylation through distinct mechanisms.. J. Neurosci..

[88] Clodfelder-Miller B.J., Zmijewska A.A., Johnson G.V.W., Jope R.S. (2006). Tau is hyperphosphorylated at multiple sites in mouse brain in vivo after streptozotocin-induced insulin deficiency.. Diabetes.

[89] Li Z., Zhang W., Sima A.A.F. (2007). Alzheimer-like changes in rat models of spontaneous diabetes.. Diabetes.

[90] Wang S., Zhou S., Min F., Ma J., Shi X., Bereczki E., Wu J. (2014). mTOR-mediated hyperphosphorylation of tau in the hippocampus is involved in cognitive deficits in streptozotocin-induced diabetic mice.. Metab. Brain Dis..

[91] Zhao Y.M., Pei J.J., Ji Z.J., Zhao Z.W., Qian Y.Y., Sheng S.L. (2003). Effect of amyloid precursor protein 17mer peptide on microtubule structure and tau protein hyperphosphorylation in hippocampal neurons of experimental diabetic mice.. Neuroreport.

[92] Wu J., Zhou S.L., Pi L.H., Shi X.J., Ma L.R., Chen Z., Qu M.L., Li X., Nie S.D., Liao D.F., Pei J.J., Wang S. (2017). High glucose induces formation of tau hyperphosphorylation via Cav-1-mTOR pathway: A potential molecular mechanism for diabetes-induced cognitive dysfunction.. Oncotarget.

[93] Martin L., Latypova X., Terro F. (2011). Post-translational modifications of tau protein: Implications for Alzheimer’s disease.. Neurochem. Int..

[94] Alquezar C., Arya S., Kao A.W. (2021). Tau Post-translational modifications: Dynamic transformers of tau function, degradation, and aggregation.. Front. Neurol..

[95] Theofilas P., Wang C., Butler D. (2021). Caspase inhibition mitigates tau cleavage and neurotoxicity in iPSC-induced neurons with the V337M MAPT mutation.. bioRxiv.

[96] Harris L.D., Jasem S., Licchesi J.D.F. (2020). The ubiquitin system in Alzheimer’s disease.. Adv. Exp. Med. Biol..

[97] Reinecke J.B., DeVos S.L., McGrath J.P., Shepard A.M., Goncharoff D.K., Tait D.N., Fleming S.R., Vincent M.P., Steinhilb M.L. (2011). Implicating calpain in tau-mediated toxicity in vivo.. PLoS One.

[98] Chen H.H., Liu P., Auger P., Lee S.H., Adolfsson O., Rey-Bellet L., Lafrance-Vanasse J., Friedman B.A., Pihlgren M., Muhs A., Pfeifer A., Ernst J., Ayalon G., Wildsmith K.R., Beach T.G., van der Brug M.P. (2018). Calpain-mediated tau fragmentation is altered in Alzheimer’s disease progression.. Sci. Rep..

[99] McMillan P.J., Kraemer B.C., Robinson L., Leverenz J.B., Raskind M., Schellenberg G. (2011). Truncation of tau at E391 promotes early pathologic changes in transgenic mice.. J. Neuropathol. Exp. Neurol..

[100] Tolkovsky A.M., Spillantini M.G. (2021). Tau aggregation and its relation to selected forms of neuronal cell death.. Essays Biochem..

[101] Zhang H., Cao Y., Ma L., Wei Y., Li H. (2021). Possible mechanisms of tau spread and toxicity in Alzheimer’s disease.. Front. Cell Dev. Biol..

[102] Corsetti V., Amadoro G., Gentile A., Capsoni S., Ciotti M.T., Cencioni M.T., Atlante A., Canu N., Rohn T.T., Cattaneo A., Calissano P. (2008). Identification of a caspase-derived N-terminal tau fragment in cellular and animal Alzheimer’s disease models.. Mol. Cell. Neurosci..

[103] Wang Y.P., Biernat J., Pickhardt M., Mandelkow E., Mandelkow E.M. (2007). Stepwise proteolysis liberates tau fragments that nucleate the Alzheimer-like aggregation of full-length tau in a neuronal cell model.. Proc. Natl. Acad. Sci. USA.

[104] Zhang H., Wei W., Zhao M., Ma L., Jiang X., Pei H., Cao Y., Li H. (2021). Interaction between Aβ and tau in the pathogenesis of Alzheimer’s disease.. Int. J. Biol. Sci..

[105] Gamblin T.C., Chen F., Zambrano A., Abraha A., Lagalwar S., Guillozet A.L., Lu M., Fu Y., Garcia-Sierra F., LaPointe N., Miller R., Berry R.W., Binder L.I., Cryns V.L. (2003). Caspase cleavage of tau: Linking amyloid and neurofibrillary tangles in Alzheimer’s disease.. Proc. Natl. Acad. Sci. USA.

[106] Yin H., Kuret J. (2006). C-terminal truncation modulates both nucleation and extension phases of τ fibrillization.. FEBS Lett..

[107] Conze C., Rierola M., Trushina N.I., Peters M., Janning D., Holzer M., Heinisch J.J., Arendt T., Bakota L., Brandt R. (2022). Caspase-cleaved tau is senescence-associated and induces a toxic gain of function by putting a brake on axonal transport.. Mol. Psychiatry.

[108] Zilka N., Filipcik P., Koson P., Fialova L., Skrabana R., Zilkova M., Rolkova G., Kontsekova E., Novak M. (2006). Truncated tau from sporadic Alzheimer’s disease suffices to drive neurofibrillary degeneration in vivo.. FEBS Lett..

[109] Zilkova M., Zilka N., Kovac A., Kovacech B., Skrabana R., Skrabanova M., Novak M. (2011). Hyperphosphorylated truncated protein tau induces caspase-3 independent apoptosis-like pathway in the Alzheimer’s disease cellular model.. J. Alzheimers Dis..

[110] Hrnkova M., Zilka N., Minichova Z., Koson P., Novak M. (2007). Neurodegeneration caused by expression of human truncated tau leads to progressive neurobehavioural impairment in transgenic rats.. Brain Res..

[111] Zimova I., Brezovakova V., Hromadka T., Weisova P., Cubinkova V., Valachova B., Filipcik P., Jadhav S., Smolek T., Novak M., Zilka N. (2016). Human truncated tau induces mature neurofibrillary pathology in a mouse model of human tauopathy.. J. Alzheimers Dis..

[112] Kim B., Backus C., Oh S., Feldman E.L. (2013). Hyperglycemia-induced tau cleavage in vitro and in vivo: a possible link between diabetes and Alzheimer’s disease.. J. Alzheimers Dis..

[113] El Khoury N.B., Gratuze M., Papon M.A., Bretteville A., Planel E. (2014). Insulin dysfunction and tau pathology.. Front. Cell. Neurosci..

[114] Santos R.X., Correia S.C., Alves M.G., Oliveira P.F., Cardoso S., Carvalho C., Duarte A.I., Santos M.S., Moreira P.I. (2014). Insulin therapy modulates mitochondrial dynamics and biogenesis, autophagy and tau protein phosphorylation in the brain of type 1 diabetic rats.. Biochim. Biophys. Acta Mol. Basis Dis..

[115] Guo C., Zhang S., Li J.Y., Ding C., Yang Z.H., Chai R., Wang X., Wang Z.Y. (2016). Chronic hyperglycemia induced via the heterozygous knockout of Pdx1 worsens neuropathological lesion in an Alzheimer mouse model.. Sci. Rep..

[116] Wei T.H., Hsieh C.L. (2020). Effect of acupuncture on the p38 signaling pathway in several nervous system diseases: A systematic review.. Int. J. Mol. Sci..

[117] Jin Y., Fan Y., Yan E., Liu Z., Zong Z., Qi Z. (2006). Effects of sodium ferulate on amyloid-beta-induced MKK3/MKK6-p38 MAPK-Hsp27 signal pathway and apoptosis in rat hippocampus.. Acta Pharmacol. Sin..

[118] Falcicchia C., Tozzi F., Arancio O., Watterson D.M., Origlia N. (2020). Involvement of p38 MAPK in synaptic function and dysfunction.. Int. J. Mol. Sci..

[119] Yamazaki Y., Zhao N., Caulfield T.R., Liu C.C., Bu G. (2019). Apolipoprotein E and Alzheimer disease: pathobiology and targeting strategies.. Nat. Rev. Neurol..

[120] Emrani S., Arain H.A., DeMarshall C., Nuriel T. (2020). APOE4 is associated with cognitive and pathological heterogeneity in patients with Alzheimer’s disease: a systematic review.. Alzheimers Res. Ther..

[121] Starks E.J., Patrick O’Grady J., Hoscheidt S.M., Racine A.M., Carlsson C.M., Zetterberg H., Blennow K., Okonkwo O.C., Puglielli L., Asthana S., Dowling N.M., Gleason C.E., Anderson R.M., Davenport-Sis N.J., DeRungs L.M., Sager M.A., Johnson S.C., Bendlin B.B. (2015). Insulin resistance is associated with higher cerebrospinal fluid tau levels in asymptomatic APOEɛ4 carriers.. J. Alzheimers Dis..

[122] Sun Y., Ma C., Sun H., Wang H., Peng W., Zhou Z., Wang H., Pi C., Shi Y., He X. (2020). Metabolism: A novel shared link between diabetes mellitus and Alzheimer’s disease.. J. Diabetes Res..

[123] Namba Y., Tomonaga M., Kawasaki H., Otomo E., Ikeda K. (1991). Apolipoprotein E immunoreactivity in cerebral amyloid deposits and neurofibrillary tangles in Alzheimer’s disease and kuru plaque amyloid in Creutzfeldt-Jakob disease.. Brain Res..

[124] Kok E., Haikonen S., Luoto T., Huhtala H., Goebeler S., Haapasalo H., Karhunen P.J. (2009). Apolipoprotein E-dependent accumulation of Alzheimer disease-related lesions begins in middle age.. Ann. Neurol..

[125] Kanekiyo T., Xu H., Bu G. (2014). ApoE and Aβ in Alzheimer’s disease: accidental encounters or partners?. Neuron.

[126] Rannikmäe K., Kalaria R.N., Greenberg S.M., Chui H.C., Schmitt F.A., Samarasekera N., Al-Shahi Salman R., Sudlow C.L.M. (2014). APOE associations with severe CAA-associated vasculopathic changes: collaborative meta-analysis.. J. Neurol. Neurosurg. Psychiatry.

[127] Biffi A., Sonni A., Anderson C.D., Kissela B., Jagiella J.M., Schmidt H., Jimenez-Conde J., Hansen B.M., Fernandez-Cadenas I., Cortellini L., Ayres A., Schwab K., Juchniewicz K., Urbanik A., Rost N.S., Viswanathan A., Seifert-Held T., Stoegerer E.M., Tomás M., Rabionet R., Estivill X., Brown D.L., Silliman S.L., Selim M., Worrall B.B., Meschia J.F., Montaner J., Lindgren A., Roquer J., Schmidt R., Greenberg S.M., Slowik A., Broderick J.P., Woo D., Rosand J. (2010). Variants at APOE influence risk of deep and lobar intracerebral hemorrhage.. Ann. Neurol..

[128] Liu C.C., Kanekiyo T., Xu H., Bu G., Bu G. (2013). Apolipoprotein E and Alzheimer disease: risk, mechanisms and therapy.. Nat. Rev. Neurol..

[129] Matsuzaki T., Sasaki K., Tanizaki Y., Hata J., Fujimi K., Matsui Y., Sekita A., Suzuki S.O., Kanba S., Kiyohara Y., Iwaki T. (2010). Insulin resistance is associated with the pathology of Alzheimer disease: The Hisayama Study.. Neurology.

[130] Bell R.D., Winkler E.A., Singh I., Sagare A.P., Deane R., Wu Z., Holtzman D.M., Betsholtz C., Armulik A., Sallstrom J., Berk B.C., Zlokovic B.V. (2012). Apolipoprotein E controls cerebrovascular integrity via cyclophilin A.. Nature.

[131] Heneka M.T., Carson M.J., Khoury J.E., Landreth G.E., Brosseron F., Feinstein D.L., Jacobs A.H., Wyss-Coray T., Vitorica J., Ransohoff R.M., Herrup K., Frautschy S.A., Finsen B., Brown G.C., Verkhratsky A., Yamanaka K., Koistinaho J., Latz E., Halle A., Petzold G.C., Town T., Morgan D., Shinohara M.L., Perry V.H., Holmes C., Bazan N.G., Brooks D.J., Hunot S., Joseph B., Deigendesch N., Garaschuk O., Boddeke E., Dinarello C.A., Breitner J.C., Cole G.M., Golenbock D.T., Kummer M.P. (2015). Neuroinflammation in Alzheimer’s disease.. Lancet Neurol..

[132] LaDu M.J., Shah J.A., Reardon C.A., Getz G.S., Bu G., Hu J., Guo L., Van Eldik L.J. (2001). Apolipoprotein E and apolipoprotein E receptors modulate Aβ-induced glial neuroinflammatory responses.. Neurochem. Int..

[133] Keene C.D., Cudaback E., Li X., Montine K.S., Montine T.J. (2011). Apolipoprotein E isoforms and regulation of the innate immune response in brain of patients with Alzheimer’s disease.. Curr. Opin. Neurobiol..

[134] Lynch J.R., Tang W., Wang H., Vitek M.P., Bennett E.R., Sullivan P.M., Warner D.S., Laskowitz D.T. (2003). APOE genotype and an ApoE-mimetic peptide modify the systemic and central nervous system inflammatory response.. J. Biol. Chem..

[135] Ringman J.M., Elashoff D., Geschwind D.H., Welsh B.T., Gylys K.H., Lee C., Cummings J.L., Cole G.M. (2012). Plasma signaling proteins in persons at genetic risk for Alzheimer disease: influence of APOE genotype.. Arch. Neurol..

[136] Boldrini M., Fulmore C.A., Tartt A.N., Simeon L.R., Pavlova I., Poposka V., Rosoklija G.B., Stankov A., Arango V., Dwork A.J., Hen R., Mann J.J. (2018). Human hippocampal neurogenesis persists throughout aging.. Cell Stem Cell.

[137] Spalding K.L., Bergmann O., Alkass K., Bernard S., Salehpour M., Huttner H.B., Boström E., Westerlund I., Vial C., Buchholz B.A., Possnert G., Mash D.C., Druid H., Frisén J. (2013). Dynamics of hippocampal neurogenesis in adult humans.. Cell.

[138] Moreno-Jiménez E.P., Flor-García M., Terreros-Roncal J., Rábano A., Cafini F., Pallas-Bazarra N., Ávila J., Llorens-Martín M. (2019). Adult hippocampal neurogenesis is abundant in neurologically healthy subjects and drops sharply in patients with Alzheimer’s disease.. Nat. Med..

[139] Mu Y., Gage F.H. (2011). Adult hippocampal neurogenesis and its role in Alzheimer’s disease.. Mol. Neurodegener..

[140] Yang C.P., Gilley J.A., Zhang G., Kernie S.G. (2011). ApoE is required for maintenance of the dentate gyrus neural progenitor pool.. Development.

[141] Najm R., Jones E.A., Huang Y. (2019). Apolipoprotein E4, inhibitory network dysfunction, and Alzheimer’s disease.. Mol. Neurodegener..

[142] Andrews-Zwilling Y., Bien-Ly N., Xu Q., Li G., Bernardo A., Yoon S.Y., Zwilling D., Yan T.X., Chen L., Huang Y. (2010). Apolipoprotein E4 causes age- and Tau-dependent impairment of GABAergic interneurons, leading to learning and memory deficits in mice.. J. Neurosci..

[143] Wu M., Zhang M., Yin X., Chen K., Hu Z., Zhou Q., Cao X., Chen Z., Liu D. (2021). The role of pathological tau in synaptic dysfunction in Alzheimer’s diseases.. Transl. Neurodegener..

[144] Sen A., Nelson T.J., Alkon D.L. (2017). ApoE isoforms differentially regulates cleavage and secretion of BDNF.. Mol. Brain.

[145] Mahley R.W., Huang Y. (2012). Apolipoprotein e sets the stage: response to injury triggers neuropathology.. Neuron.

[146] Ji Y., Gong Y., Gan W., Beach T., Holtzman D.M., Wisniewski T. (2003). Apolipoprotein E isoform-specific regulation of dendritic spine morphology in apolipoprotein E transgenic mice and Alzheimer’s disease patients.. Neuroscience.

[147] Dumanis S.B., Tesoriero J.A., Babus L.W., Nguyen M.T., Trotter J.H., Ladu M.J., Weeber E.J., Turner R.S., Xu B., Rebeck G.W., Hoe H.S. (2009). ApoE4 decreases spine density and dendritic complexity in cortical neurons in vivo.. J. Neurosci..

[148] Sen A., Alkon D.L., Nelson T.J. (2012). Apolipoprotein E3 (ApoE3) but not ApoE4 protects against synaptic loss through increased expression of protein kinase C epsilon.. J. Biol. Chem..

[149] Klein R.C., Mace B.E., Moore S.D., Sullivan P.M. (2010). Progressive loss of synaptic integrity in human apolipoprotein E4 targeted replacement mice and attenuation by apolipoprotein E2.. Neuroscience.

[150] Chen Y., Durakoglugil M.S., Xian X., Herz J. (2010). ApoE4 reduces glutamate receptor function and synaptic plasticity by selectively impairing ApoE receptor recycling.. Proc. Natl. Acad. Sci. USA.

[151] Han W., Li C. (2010). Linking type 2 diabetes and Alzheimer’s disease.. Proc. Natl. Acad. Sci. USA.

[152] Crane P.K., Walker R., Hubbard R.A., Li G., Nathan D.M., Zheng H., Haneuse S., Craft S., Montine T.J., Kahn S.E., McCormick W., McCurry S.M., Bowen J.D., Larson E.B. (2013). Glucose levels and risk of dementia.. N. Engl. J. Med..

[153] Akimoto H., Negishi A., Oshima S., Wakiyama H., Okita M., Horii N., Inoue N., Ohshima S., Kobayashi D. (2020). Antidiabetic drugs for the risk of Alzheimer disease in patients with type 2 DM using FAERS.. Am. J. Alzheimers Dis. Other Demen..

[154] Kazmi M., Zaib S., Ibrar A., Amjad S.T., Shafique Z., Mehsud S., Saeed A., Iqbal J., Khan I. (2018). A new entry into the portfolio of α-glucosidase inhibitors as potent therapeutics for type 2 diabetes: Design, bioevaluation and one-pot multi-component synthesis of diamine-bridged coumarinyl oxadiazole conjugates.. Bioorg. Chem..

[155] Santos C.M.M., Freitas M., Fernandes E. (2018). A comprehensive review on xanthone derivatives as α-glucosidase inhibitors.. Eur. J. Med. Chem..

[156] Wachters-Hagedoorn R.E., Priebe M.G., Heimweg J.A.J., Heiner A.M., Elzinga H., Stellaard F., Vonk R.J. (2007). Low-dose acarbose does not delay digestion of starch but reduces its bioavailability.. Diabet. Med..

[157] Yan W.W., Chen G.H., Wang F., Tong J.J., Tao F. (2015). Long-term acarbose administration alleviating the impairment of spatial learning and memory in the SAMP8 mice was associated with alleviated reduction of insulin system and acetylated H4K8.. Brain Res..

[158] Manandhar S., Priya K., Mehta C.H., Nayak U.Y., Kabekkodu S.P., Pai K.S.R. (2021). Repositioning of antidiabetic drugs for Alzheimer’s disease: possibility of Wnt signaling modulation by targeting LRP6 an in silico based study.. J. Biomol. Struct. Dyn..

[159] Zhou J.B., Tang X., Han M., Yang J., Simó R. (2020). Impact of antidiabetic agents on dementia risk: A Bayesian network meta-analysis.. Metabolism.

[160] Proença C., Freitas M., Ribeiro D., Oliveira E.F.T., Sousa J.L.C., Tomé S.M., Ramos M.J., Silva A.M.S., Fernandes P.A., Fernandes E. (2017). α-Glucosidase inhibition by flavonoids: an in vitro and in silico structure-activity relationship study.. J. Enzyme Inhib. Med. Chem..

[161] Wu Y., Liu W., Yang T., Li M., Qin L., Wu L., Liu T. (2021). Oral administration of mangiferin ameliorates diabetes in animal models: a meta-analysis and systematic review.. Nutr. Res..

[162] Shi G.J., Li Y., Cao Q.H., Wu H.X., Tang X.Y., Gao X.H., Yu J.Q., Chen Z., Yang Y. (2019). In vitro and in vivo evidence that quercetin protects against diabetes and its complications: A systematic review of the literature.. Biomed. Pharmacother..

[163] Penumala M., Zinka R.B., Shaik J.B., Amooru Gangaiah D. (2017). In vitro screening of three Indian medicinal plants for their phytochemicals, anticholinesterase, antiglucosidase, antioxidant, and neuroprotective effects.. BioMed Res. Int..

[164] Silva A.P.G.S., De Jesus A.R.X., Martins A.I.M. (2015). New c-glycosylpolyphenol antidiabetic agents, effect on glucose tolerance and interaction with beta-amyloid. therapeutic applications of the synthesized agent (s) and of genista tenera ethyl acetate extracts containing some of those agents..

